# Effect of Drought Stress and Developmental Stages on Microbial Community Structure and Diversity in Peanut Rhizosphere Soil

**DOI:** 10.3390/ijms20092265

**Published:** 2019-05-08

**Authors:** Liangxiang Dai, Guanchu Zhang, Zipeng Yu, Hong Ding, Yang Xu, Zhimeng Zhang

**Affiliations:** 1 Shandong Peanut Research Institute, Shandong Academy of Agricultural Sciences, Qingdao 266100, China; liangxiangd@163.com (L.D.); guanchuzhang@126.com (G.Z.); dingpeanut@163.com (H.D.); 2 State Key Laboratory of Crop Biology, Shandong Agricultural University, Tai’an 271018, China; yzp52120090916@163.com

**Keywords:** peanut (*Arachis hypogaea* L.), rhizosphere, microbial community structure, drought stress

## Abstract

Background: Peanut (*Arachis hypogaea* L.), an important oilseed and food legume, is widely cultivated in the semi-arid tropics. Drought is the major stress in this region which limits productivity. Microbial communities in the rhizosphere are of special importance to stress tolerance. However, relatively little is known about the relationship between drought and microbial communities in peanuts. Method: In this study, deep sequencing of the V3-V4 region of the 16S rRNA gene was performed to characterize the microbial community structure of drought-treated and untreated peanuts. Results: Taxonomic analysis showed that Actinobacteria, Proteobacteria, Saccharibacteria, Chloroflexi, Acidobacteria and Cyanobacteria were the dominant phyla in the peanut rhizosphere. Comparisons of microbial community structure of peanuts revealed that the relative abundance of Actinobacteria and Acidobacteria dramatically increased in the seedling and podding stages in drought-treated soil, while that of Cyanobacteria and Gemmatimonadetes increased in the flowering stage in drought-treated rhizospheres. Metagenomic profiling indicated that sequences related to metabolism, signaling transduction, defense mechanism and basic vital activity were enriched in the drought-treated rhizosphere, which may have implications for plant survival and drought tolerance. Conclusion: This microbial communities study will form the foundation for future improvement of drought tolerance of peanuts via modification of the soil microbes.

## 1. Introduction

Peanut or groundnut (*Arachis hypogaea* L., Fabaceae), one of the five most important oilseed crops, serves as a good source of protein, calories, vitamins and minerals [1,2]. It is consumed both as oilseed and livestock fodder, forming an important revenue source for farmers as well as for commercial producers. Peanut is known to be more tolerant to drought stress than most other related plant species [3]. About 60% of the world peanut production comes from the semi-arid tropics such as Africa, Asia, North, and South America [4,5]. However, extreme drought conditions in these regions still affect productivity and quality of peanuts, which can cause an annual loss of 6 million tons worth about 520 million USD [6,7]. Investigating the molecular mechanism of drought stress response and further improving drought tolerance of peanuts would be of great significance to the peanut industry.

Drought stress causes hyperosmotic stress characterized by decreased turgor pressure and water loss, resulting in membrane disorganization, the inhibition of photosynthesis, the accumulation of reactive oxygen species and ultimately, cell and plant death [8]. To survive drought stress, plants must respond properly through a variety of molecular mechanisms, including morphological adaptations, physiological acclimation, and cellular adjustment [9]. Morphological adaptations make use of the developmental plasticity of plants via changing cell or tissue shape and size, and physiological acclimation involves increased water uptake and decreased water loss, while cellular adjustment applies to enhance osmoprotection, antioxidative capacity and desiccation tolerance [10,11,12]. In recent years, various studies have emerged implicating the root external environment (rhizosphere) also to be involved in a wide range of stress tolerances in plants, including high salinity, drought and pathogen infection, which provides a novel direction in future improvement of stress tolerance of peanuts via modifying the soil microbial community [13,14,15,16]. However, studies of the peanut rhizosphere are scarce.

The rhizosphere is a “hot spot” of intense microbial activity in the soil surrounding the roots, and is the focus in the field of plant stress response [17]. Plant roots exude various chemicals and nutrients into the rhizosphere and attract a variety of microbes, such as bacteria, fungi, algaes, and protozoa [17,18]. Rhizosphere-associated microbes possess diverse metabolic capabilities and play crucial roles in the rhizosphere ecosystem, including nutrient cycling and organic matter decomposition, which exert positive effects on plants’ health and growth [19]. More importantly, the microbial community can also play a crucial role in plant growth and adaptation to various environmental stresses [20,21,22]. Over the past decade, various studies have emerged implicating members of the microbial community in enhancing plants stress tolerance by providing a buffer zone for plants against stress, producing various plant growth promoting hormones and enhancing nutrient availability [18,23,24]. The changed microbial community during stress may, at least in part, have implications for plant survival and health. There is a need to identify root-associated microbial communities that thrive under adverse environments and can confer stress tolerance and potentially be advantageous to the host.

The root microbial community structure differs across plant species, the stages of plant development, soil types and agricultural management. In this study, we examined the impacts of developmental stages on the drought-treated root-associated microbial community structure of cultivated peanuts. By high-throughput sequencing of the 16S rRNA genes of the peanut rhizosphere microbial genomes generated in normal and drought conditions during various developmental stages, the composition of the microbial community and its effect on drought response in peanuts were evaluated. The study aimed to assess and provide new insight into the influence of developmental stages and drought stress on the composition of the microbial community in the peanut rhizosphere. 

## 2. Results 

### 2.1. Overall Sequence Data of Microbial Communities in the Peanut Rhizosphere

To explore the microbial shift of the peanut rhizosphere under drought stress conditions, 16S rRNA gene sequencing was performed to sequence the bacterial genomes under normal and drought conditions. We combined controlled root surface and rhizospheric soil to control rhizosphere (CR), the various drought-treated root surface and rhizospheric soils were defined as seedling stage drought-treated rhizosphere (SDR), flowering stage drought-treated rhizosphere (FDR) and podding stage drought-treated rhizosphere (PDR), respectively. Low quality reads were filtered using the Quantitative Insights into Microbial Ecology software (QIIME) and trimming the primers, adapters and barcodes, sequencing quantities of each sample counted were listed in Supplementary Table S1. In total, 1,731,115 high quality clean reads passed quality screening and most of the sequence lengths were between 400–450 bp (Supplementary Figure S1A and Table S1). Operational taxonomic units (OTUs) were generated with 97% sequence similarity. A total of 3639 OTUs were found in rhizosphere soil samples, in which 2891 OTUs were shared by all the soil groups, while 22, 30, 37, and 60 OTUs were only present in CR, SDR, FDR, and PDR respectively (Supplementary Figure S1B).

### 2.2. Alpha Diversity Analysis

**Figure 1:**
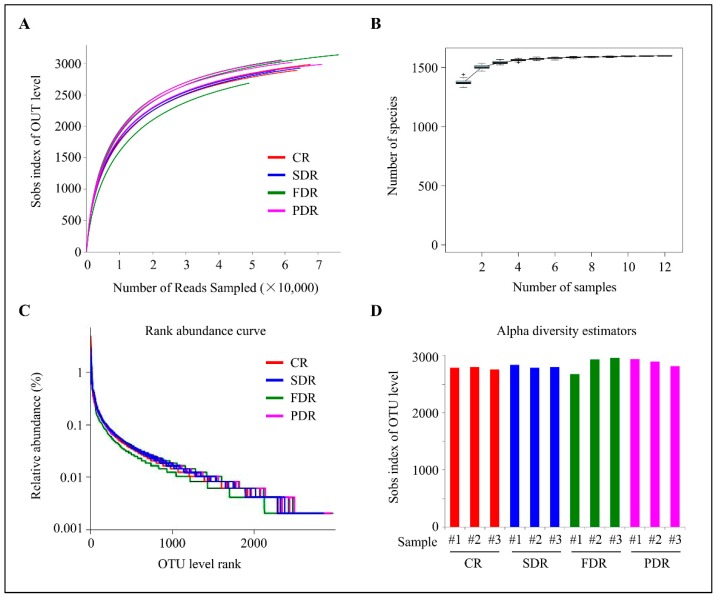
Alpha diversity analysis. (**A**) Rarefaction curve analysis showing the depth of 16S rRNA gene sequencing of peanut rhizosphere and the possibility of observing microbial community diversity. (**B**) Species accumulation curves showing the rate of increase of new species with the increase in sample size. (**C**) Rank abundance curve showing the relative species abundance and evenness. The length of the polyline on the horizontal axis reflects the number of operational taxonomic units (OTUs) in the sample and represents the richness of the microbial community. The flatness of the polyline reflects the evenness of the microbial community composition. (**D**) OTU levels via sobs index analysis showing the relative species abundance of four soil groups in peanut rhizosphere. #1, #2 and #3 represent three duplicate samples in per soil group.

Community richness and diversity of the microbial ecosystem were examined via alpha diversity analysis. Rarefaction curve analysis exhibited a high depth of 16S rRNA gene sequencing and a great possibility of observing community diversity in each peanut rhizosphere (Figure 1A). In the species accumulation curves, the increase rate of new species followed with the increase in sample size during the sampling process, implying that the sequencing depth was high enough to observe community richness (Figure 1B). Rank abundance curves showed that all the four soil groups had high species evenness and homogeneity (Figure 1C). 

Many other indices can also reflect the alpha diversity of the microbial community. Sobs exhibits the numbers of OTUs in the peanut rhizosphere, and coverage reflects the sequencing depth to observe community richness. Chao1 and ace reflect the community abundance, and the Shannon and Simpson indices exhibit community diversity (Supplementary Table S2). The diversity indices indicated that the four soil groups in peanut rhizosphere had basically similar species richness and diversity (Figure 1D and Supplementary Table S2).

### 2.3. Rhizosphere Microbial Community Structure

**Figure 2:**
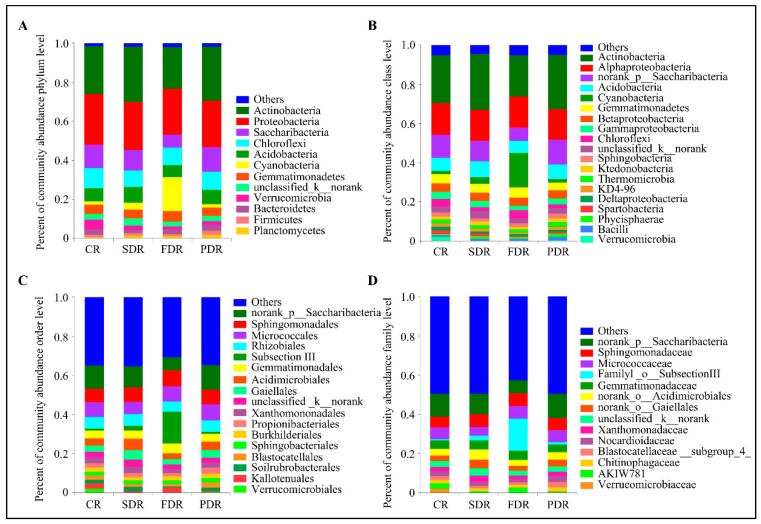
Microbial community structure in peanut rhizosphere at the phylum, class, order and family level. (**A**) Percent of microbial community abundance at the phylum level in four soil groups in peanut rhizosphere. The relative abundance is calculated by averaging the abundances of duplicate samples in each soil group in peanut rhizosphere. (**B**) Percent of microbial community abundance at the class level in four soil groups in peanut rhizosphere. The relative abundance is calculated by averaging the abundances of duplicate samples. (**C**) Percent of microbial community abundance at the order level in four soil groups in peanut rhizosphere. The relative abundance is calculated by averaging the abundances of duplicate samples. (**D**) Percent of microbial community abundance at the family level in four soil groups in peanut rhizosphere. The relative abundance is calculated by averaging the abundances of duplicate samples. The names of “norank” and “unidentified” are all unidentified species obtained directly from database via sequence alignment.

To further analyze the microbial community structure, the abundance distributions of each sample at five levels of classification (phylum, class, order, family, and genus) were developed. The average relative abundances of four soil groups were classified into 31 phyla, but only 11 phyla were found at a relative abundance of >1% (Supplementary Table S3). Although the abundance of each phylum varied in peanut rhizosphere soil samples with drought treatment during different developmental stages, Actinobacteria, Proteobacteria, Saccharibacteria, Chloroflexi, Acidobacteria, and Cyanobacteria were the six phyla dominant in the peanut rhizosphere of drought-treated soils and untreated soils, accounting for about 80% of all microbial taxa (Figure 2A). The abundance of Cyanobacteria and Planctomycetes increased, while Proteobacteria, Chloroflexi and Verrucomicrobia decreased in three drought-treated soil groups compared to CR. In addition, SDR and PDR had distinct bacterial communities compared to FDR: The relative abundance of Actinobacteria and Acidobacteria dramatically increased in SDR and PDR, while that of Cyanobacteria and Gemmatimonadetes increased in FDR (Figure 2A). Cyanobacteria and Gemmatimonadetes were dominant phyla in FDR, and unclassified_k__norank dominated in SDR. Planctomycetes and Firmicutes were more abundant in PDR than in FDR and SDR, suggesting that the developmental stages also affected the structure of the microbial community in the rhizosphere of drought-treated plants (Figure 2A).

At the class level, most of the bacteria belonged to Actinobacteria, Alphaproteobacteria, norank_p__Saccharibacteria, Acidobacteria, Cyanobacteria, Gemmatimonadetes and Betaproteobacteria (Supplementary Table S4). In FDR, Cyanobacteria was the most abundant class, and unclassified_k__norank dominated in SDR at the class level. In addition, the dominant microbes in the phyla of Planctomycete and Firmicutes were classes of Phycisphaerae and Bacilli in PDR, respectively (Figure 2B). Compared to soil without drought treatment, the abundance of Gammaproteobacteria and Deltaproteobacteria at the phylum of Proteobacteria and Verrucomicrobiae at the phylum of Verrucomicrobia decreased in all drought-treated soils (Figure 2B).

Norank_p__Saccharibacteria was the most abundant order, Sphingomonadales and Subsection III were the dominant orders in FDR, and Propionibacteriales and Blastocatellales had a higher relative abundance in PDR. Acidimicrobiales, unclassified_k__norank and Xanthomononadales significantly increased in SDR compared with that in CR (Figure 2C). At the family level, norank_p__Saccharibacteria, Sphingomonadaceae, Micrococcaceae, Familyl_o__Subsection III, Gemmatimonadaceae and norank_o__Acidimicrobiales were the abundant families in the four soil groups of peanut rhizosphere. The abundance of Familyl_o__Subsection III was higher in FDR, and Nocardioidaceae and Blastocatellaceae__Subgroup_4_ were relatively higher in PDR, whereas norank_o__Acidimicrobiales, norank_o__Gaiellales and Xanthomonadaceae were prominent in SDR (Figure 2D).

**Figure 3:**
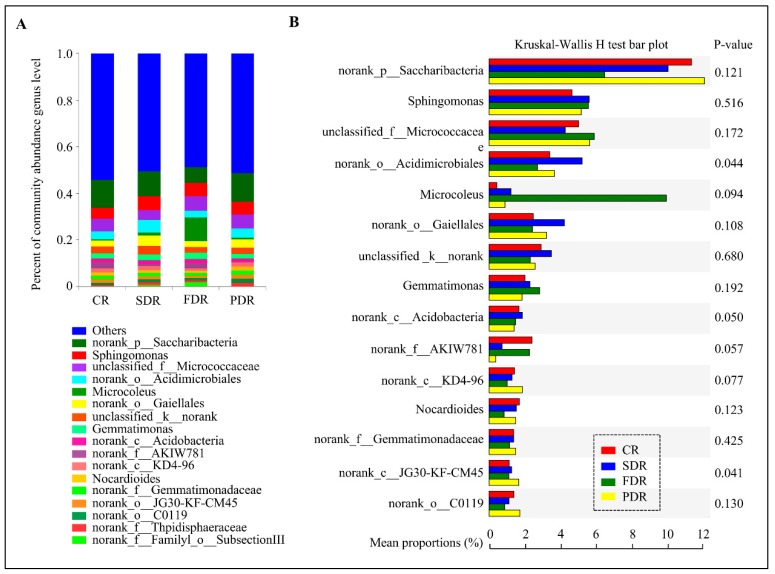
Microbial community structure and diversity in drought-treated and untreated peanut rhizosphere. (**A**) Percent of microbial community abundance at the genus level in four soil groups in peanut rhizosphere. The relative abundance is calculated by averaging the abundances of duplicate samples. (**B**) Microbial community diversity in drought-treated and untreated peanut rhizosphere via Wilcoxon rank-sum test bar plot. Left image represents the proportions of various genera in four soil groups. Right image represents the difference between proportions in 95% confidence intervals. The names of “norank” and “unidentified” are all unidentified species obtained directly from database via sequence alignment.

A thorough investigation at the genus level showed that 503 taxa were classified from the four rhizosphere communities, whereas most of genera were <15%, implying high microbial diversity in the four soil groups (Supplementary Excel S1). As shown in Figure 3A, *norank_p__Saccharibacteria*, *Sphingomonas*, *unclassified_f__Micrococcaceae*, *norank_o__Acidimicrobiales*, *Microcoleus* and *norank_o__Gaiellales* were predominantly found in the rhizosphere soil, belong to the phylum of Saccharibacteria, Proteobacteria, Actinobacteria, Actinobacteria, Cyanobacteria and Actinobacteria, respectively. Drought treatment increased the abundance of *Sphingomonas* and *Streptomyces* but reduced the abundance of *norank_c__KD4-96* (Figure 3A and Supplementary Table S5). The root microbial community structure also differed across the stages of plant development. *Microcoleus* was the dominant genus in FDR, while *norank_p__Saccharibacteria* had a higher relative abundance in PDR. *Norank_o__Acidimicrobiales* and *norank_o__Gaiellales* dominated in SDR (Figure 3A). Furthermore, the generic diversity among four soil groups was clearly demonstrated by Wilcoxon rank-sum test, and the most predominant genus in four soil groups was *norank_p__Saccharibacteria*. In addition, *Sphingomonas* was the dominant genus in all drought-treated soil groups and *Microcoleus* dominated in FDR (Figure 3B and Supplementary Figure S2). 

### 2.4. Clustering Analysis of Rhizosphere Microbial Community Composition

In order to observe the similarities and dissimilarities among four soil groups, principal component analysis (PCA), principal co-ordinates analysis (PCoA) and clustering analysis were performed. 

**Figure 4:**
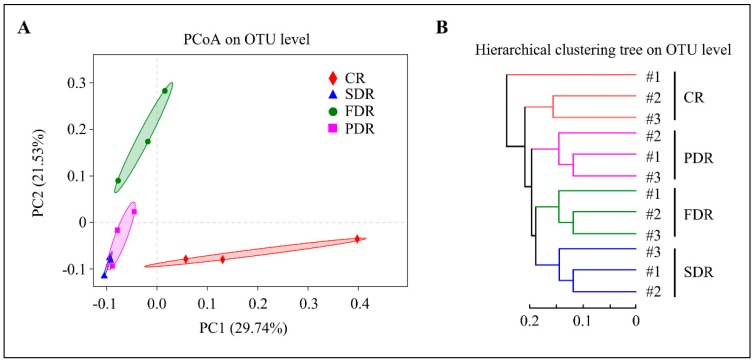
Beta diversity analysis. (**A**) Principal co-ordinates analysis (PCoA) analysis. The same color points belong to the same soil group, and the same soil group points are marked by ellipses. The samples belonging to the same soil group are closer to each other and the samples from different soil groups are farther apart. (**B**) Hierarchical clustering is clustered according to groups’ similarity. The branch length among soil groups represent the degree of similarity among the four soil groups. #1, #2 and #3 represent three duplicate samples in per soil group.

Distinct differences in microbial communities existed among four soil groups. The first two principal components (PC1 and PC2) of PCA explained 44.38% and 19.4% of the total variation, respectively (Supplementary Figure S3A). PCoA analysis also showed that the microbial community structures of four soil groups were very different and the two principal components (PC1 and PC2) of PCoA explained 29.74% and 21.53%, respectively (Figure 4A). The analysis of similarities among and within the soil groups was also determined. There was a significant bray_curtis distance among different soil groups, whereas SDR-SDR, FDR-FDR and PDR-PDR had very little bray_curtis distance in the analysis of similarities (Supplementary Figure S3B). Cluster analysis revealed that the microbial community structures of various soil groups were diverse and farther apart from each other as other beta analyses, whereas the duplicate samples in SDR, FDR, PDR and CR were similar and clustered together (Figure 4B).

**Figure 5:**
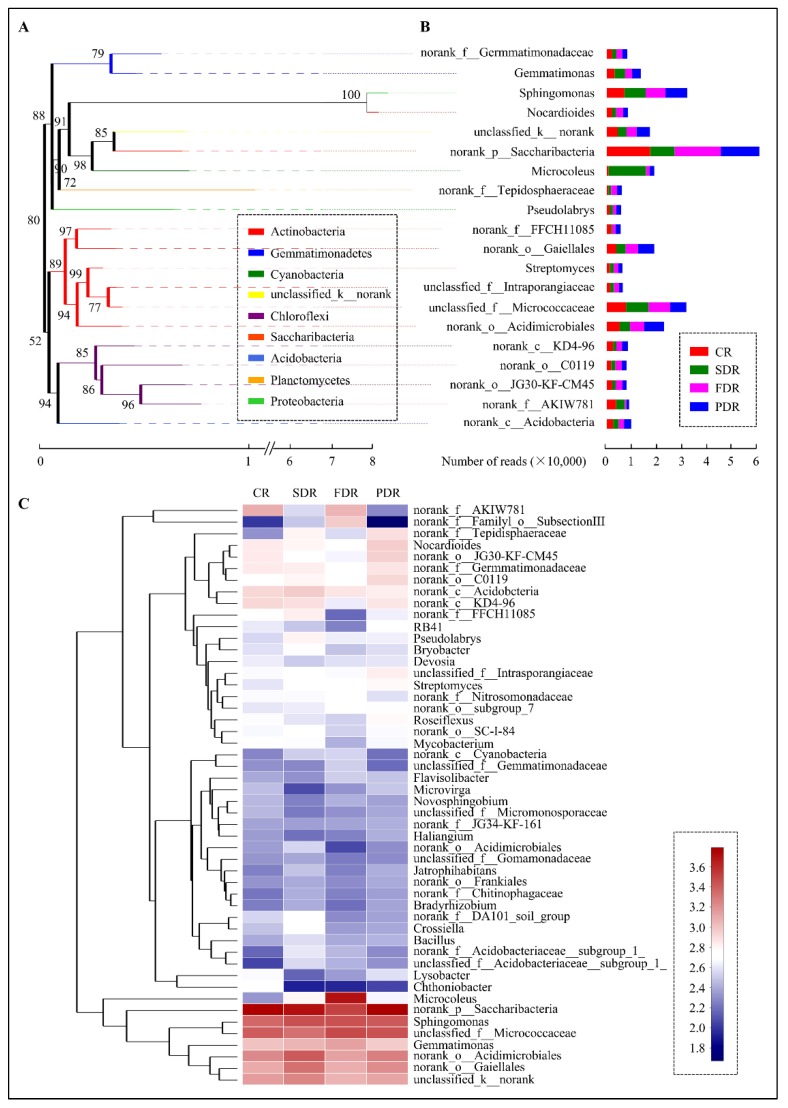
Taxonomic analysis through phylogenetic tree and heat map. (**A**) A phylogenetic tree showing the relationship among drought-treated and untreated soil groups. The phylogenetic tree was constructed on the basis of 16S rRNA gene sequences. Bootstrap values were obtained from a search with 1000 replicates and are shown at the nodes. Species names and proportions in four soil groups showing in the right. (**B**) The heat map visualization with hierarchical clustering of the top 50 most abundant genera was generated according to the similarity among their constituents, and were arranged in a horizontal order according to the clustering results. (**C**) In the figure, red represents the more abundant genera in the corresponding soil group, and blue represents the less abundant genera. The names of “norank” and “unidentified” are all unidentified species obtained directly from database via sequence alignment.

The top 20 most abundant genera belonged to the phyla of Actinobacteria, Saccharibacteria and Chloroflexi in peanut rhizosphere (Figure 5A,B and Supplementary Figure S4). In addition, the most predominant genus was *norank_p__Saccharibacteria* in all the soil groups (Figure 5A,B). 

In order to check the distinct differences in microbial community structure among the four soil groups, the community composition data for each taxonomic level were clustered on the basis of the abundance distribution of taxa or the degree of similarity among the soil groups through heat maps. Through clustering, high and low abundance taxa of top 50 most abundant genera were distinguished among the four soil groups. *Sphingomonas*, *Streptomyces* and *norank_f__Tepidisphaeraceae*, at genus level were relatively abundant in the peanut rhizosphere under drought stress conditions as compared to the control but reduced the abundance of *Chthoniobacter* and *norank_c__KD4-96* (Figure 5C).

### 2.5. Specific Phylotypes of Peanut Rhizosphere Modulate by Drought Stress

**Figure 6:**
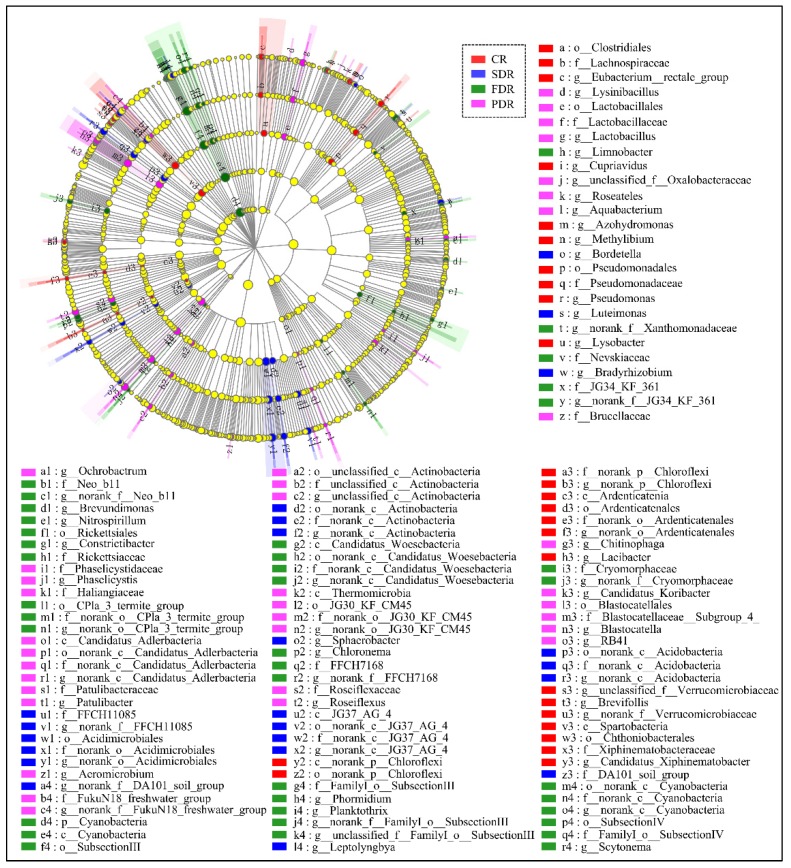
Cladogram showing specific phylotypes of peanut rhizosphere responding to drought stress. Indicator bacteria with linear discriminant analysis (LDA) scores of 3 or greater in microbial communities associated with soil from drought-treated and untreated soil groups. Circles indicate phylogenetic levels from phylum to genus (from the inner circle to the outer circle). The diameter of each circle is proportional to the abundance of the group. The names of “norank” and “unidentified” are all unidentified species obtained directly from database via sequence alignment.

As an algorithm for high-dimensional biomarker discovery and explanation of genomic features characterizing the statistically different among four soil groups, linear discriminant analysis (LDA) effect size (LEfSe) was employed to compare microbial communities and identify specific phylotypes of peanut rhizosphere responding to drought stress. Statistical analysis was performed from the phylum to the genus level in cladograms, and LDA scores of 3 or greater were confirmed by LEfSe (Figure 6 and Supplementary Figure S5). In SDR, four groups of microbes were significantly enriched, namely norank_c__Actinobacteria (from order to genus), Acidimicrobiales (from order to genus), norank_c__Acidobacteria (from order to genus), and JG37_AG_4 (from class to genus) (Figure 6). In FDR, the microbe Cyanobacteria (from phylum to family) and *norank_f__FamilyI_o__SubsectionIII* (genus) were evidently abundant (Figure 6). Three groups of microbes from order to genus were detected to be dramatically enriched in PDR, namely JG30_KF_CM45, Blastocatellales and unclassified_c__Actinobacteria. On the contrary, three groups of microbes were detected to be predominant in CR, namely Chloroflexi (from class to genus), Pseudomonadales (from order to genus), and Spartobacteria (from class to genus) (Figure 6). All the results indicate that dominant microbes during different developmental stages are distinct, which may be favorable for special drought resistance mechanisms in peanuts. 

### 2.6. Metabolic Functional Features of the Microbial Community Present in Peanut Rhizosphere

**Figure 7:**
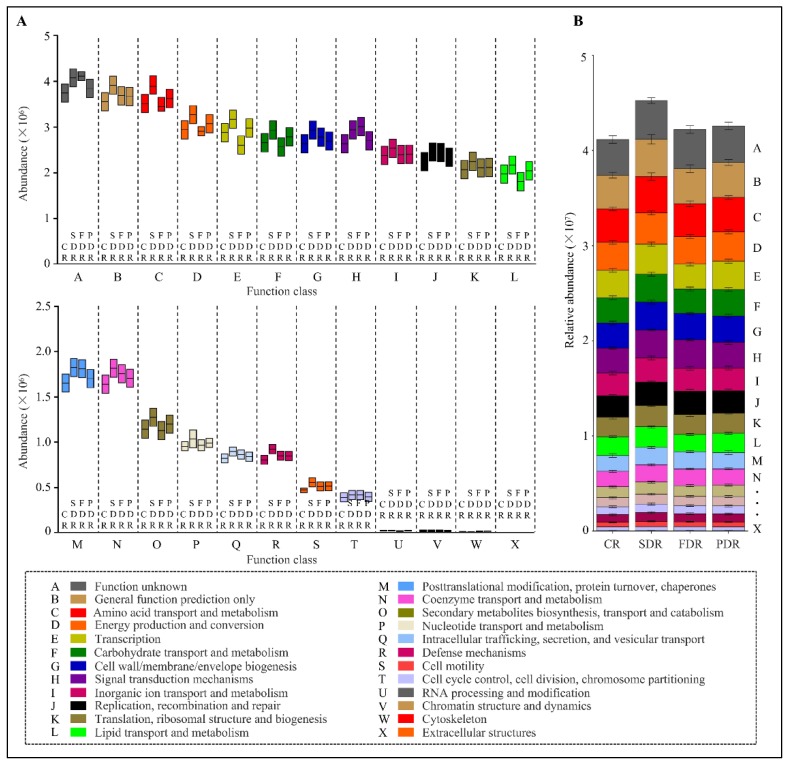
The microbial functional features in three drought-treated soil groups via Cluster of Orthologous Groups (COG) analysis. (**A**) Box-plot showing the relative abundance and diversity of various functional groups in drought-treated and untreated soil groups. (**B**) Bar chart showing the relative abundance and diversity of functional groups in drought-treated and untreated soil groups. The relative abundance is calculated by averaging the abundances of duplicate samples. Different COG groups were displayed in different colors in the bottom.

To better understand the important role of the microbial community isolated from peanut rhizosphere, the PICRUSt10 (phylogenetic investigation of communities by reconstruction of unobserved states) program was used to predict functional features of microbial community via 16S rRNA gene amplification based high-throughput sequencing data in the context of the Cluster of Orthologous Groups (COG) database. Some metabolic functions (nucleotide transport and metabolism and coenzyme transport and metabolism) and basic vital activities (cell cycle control, cell division, chromosome partitioning; translation, ribosomal structure and biogenesis; replication, recombination and repair; cell motility; cell wall/membrane/envelope biogenesis; posttranslational modification, protein turnover, chaperones) were enriched in all the drought-treated soil groups, which implies that the microbial metabolism and vital activity tended to be vigorous in the drought-treated samples (Figure 7A,B). Encouragingly, signal transduction mechanism and defense mechanism in microbial community of drought-treated soils were also significantly higher than that in CR (Figure 7A). These vigorous function groups may be related to the stress response of microbial community in the drought-treated soil groups.

## 3. Discussion

Plants root exudation attracts microbes from the nearby soil environment and affects the microbial community structure of the rhizosphere microbiome by changing the physical and chemical properties of soil [25]. In the present work, the peanut microbial community diversity and structure under normal conditions and drought stress during various developmental stages were examined via high-throughput sequencing of the 16S rRNA genes. Although the abundance of each phylum is different among various soil groups, Actinobacteria, Proteobacteria, Saccharibacteria, Chloroflexi, Acidobacteria, and Cyanobacteria were the six phyla dominant in the peanut rhizosphere of drought-treated and untreated soils (Figure 2). In the previous study, Acidobacteria, Actinobacteria, Bacteroidetes, Cyanobacteria, Firmicutes and Proteobacteria dominated in rhizosphere soil of barley [26]. Qiao et al. studied rhizosphere microbial communities in nutrient-rich soil and cotton continuous cropping field soil and found that Acidobacteria, Actinobacteria, Bacteroidetes, Planctomycetes, Proteobacteria and Verrucomicrobia were more abundant than other microbes [27]. Seven microbial phyla, namely Proteobacteria, Bacteroidetes, Actinobacteria, Acidobacteria, Firmicutes, Gemmatimonadetes and Cyanobacteria dominated in rhizosphere soil of Arabidopsis [28]. Although dominant microbial phyla in rhizospheres varies among different plants, Actinobacteria, Acidobacteria and Proteobacteria are the mutually dominant microbial phyla of peanuts and the above plants, indicating that they may be the most common dominant microbial phyla in plant rhizospheres. In addition, compared with other plants, Saccharibacteria and Chloroflexi were more abundant in the rhizosphere soil of peanuts, which may be a result of specific root exudates of peanuts.

Many biotic and abiotic stresses also alter rhizosphere microbial community structures, because some microbial communities can sense plant signal molecules under stresses, which can trigger some microbial populations to increase or decrease [13,29]. In addition, the community composition of the rhizosphere microbiome is also affected by the stages of plant development [30,31]. Our results demonstrated that drought stress and developmental stages can both quickly lead to the shift of microbial community and the enrichment of specific microbial species in peanut rhizosphere (Figure 2, Figure 3, Figure 4 and Figure 5). The abundance of Cyanobacteria and Planctomycetes increased in three drought-treated soil groups compared to CR. Cyanobacteria and Gemmatimonadetes were dominant phyla in FDR, and unclassified_k__norank dominated in SDR. Planctomycetes and Firmicutes were more abundant in PDR than in FDR and SDR (Figure 2A). In addition to the unidentified species, unclassified_k__norank, most of the drought-induced microbial communities in plant growth promotion under drought stress have been documented. Cyanobacteria can improve soil environments and survive in arid soil via accumulating soil carbon and nitrogen [32,33]. It has been well documented that Gemmatimonadetes as aerobic/anaerobic thermophilic bacteria grow well in drought conditions [34]. Phylum Firmicutes are enriched in the contaminated sites and they play various roles in bioremediation and stress tolerance [35]. Planctomycetes, an important anaerobic ammonium oxidation group, participate in the carbon cycle and mineral enrichment, which may be beneficial to plants via improving the concentration of available nutrients in drought-treated soils [36]. Therefore, we suggest that the diversely dominant microbes at each stage may represent the microbial taxa that are required for the growth and drought stress tolerance of the plants during corresponding developmental stages. It is of interest to identify the most favorable microbial species during various development stages in the hope of developing microbial inocula to deploy to increase stress resistance.

By studying the predicted function features of the microbial community, we found that some metabolic functions (nucleotide transport and metabolism; coenzyme transport and metabolism; and lipid transport and metabolism) were predicted to be higher in most drought-treated soil groups. Among them, the relatively higher abundance of lipid transport and metabolism in drought-treated soil groups (SDR and PDR) may be favorable to drought tolerance due to the roles lipids play as essential components of microbial membranes in environmental stresses response and survival [37]. Moreover, some basic biological processes (cell cycle control, cell division, chromosome partitioning; translation, ribosomal structure and biogenesis; replication, recombination and repair; cell wall/membrane/envelope biogenesis; protein turnover, chaperones and posttranslational modification) were enriched in drought-treated soil groups (Figure 7). Cell wall/membrane/envelope biogenesis in microbes may enhance drought and salt stress tolerance by regulating H^+^-ATPase of the plasma membrane, and these can enhance plant growth and drought tolerance as plant growth-promoting bacteria [38,39]. Some chaperones, such as heat shock proteins, are known as drought tolerance enhancers for plants [40,41]. Thus the vigorous microbial metabolism and basic biological processes in the drought-treated samples may enhance peanut stress tolerance. In addition, higher signal transduction mechanism and defense mechanism were also detected in the drought-treated soil groups, which can confer high tolerance levels to stress and toxic compounds [42]. All in all, we speculate that these pathways in microbes may have implications for plant survival and drought tolerance to some extent, which need further research. Managing rhizosphere microbes and maintaining the balance of beneficial and harmful microbes in the soil are crucial to the effectiveness of cropping practices.

## 4. Materials and Methods

### 4.1. Plant Materials and Soil Collection

Peanuts Huayu25 (cultivated peanut) were cultivated in a greenhouse at the Laixi experimental station, China (120.53°E, 36.86°N), in 2018–2019 under the conditions of 26 °C and approximately 16/8 h light/dark photoperiod. In order to characterize the microbial community in field conditions and further perform drought stress treatment, fine peanuts were grown in a transparent acrylic tank (36 cm in diameter and 26 cm tall) with tiny holes in the bottom containing the same weight of topsoil. The topsoil was dug by hand from a peanut field from the Laixi experimental station. Then the soil was dried under sunlight for a month and sieved with a 1 cm sieve, and 18 kg soil was added to each tank in the greenhouse conditions at the Laixi experimental station (Qingdao, China). The physiochemical properties of soil were examined before being added to the tank (pH 7.7, organic content 13.23 g·kg^−1^, total nitrogen 1.70 g·kg^−1^, available phosphorous 11.7 mg·kg^−1^, available potassium 103.2 mg·kg^−1^). Six full peanut seeds were planted in each tank, and watered every other day to keep the soil water content at 85% of field capacity as the previous study [43].

### 4.2. Drought Stress Treatment

After cultivating peanuts until the seedling stage, drought stress treatment was imposed on the peanuts by maintaining the soil water level at 45% field capacity for about 10 days until half of the plants exhibited drought stress symptoms (leaf curling, wilting and senescence) [43]. As a control, pots containing plants were regularly irrigated every other day to keep the soil water content at 85% of field capacity as the previous study [43]. Plants and soil samples were collected and stored in liquid nitrogen. In addition, after cultivating peanuts until the flowering and podding stages, the identical experiments were also performed. All the experiments were performed with three replicates and each soil group contained three duplicate samples.

### 4.3. Sample of Rhizosphere Compartments Collection and DNA Extraction

Rhizosphere compartments were collected as described by previous study [44]. Rhizosphere soil samples were composite samples of root surface soil and soil around the roots. Briefly, harvested roots were vigorously shaken to remove loose soil in accordance with the previous study [18]. For consistency, 5 cm of root immediately below the root-shot junction were cut off by sterile scissors from drought and control plants, and then placed in 50 mL of sterile centrifuge tube containing 40 mL PBS buffer (pH 7.0, per liter 6.33 g of NaH_2_PO_4_·H_2_O, 16.5 g of Na_2_HPO_4_·7H_2_O, 200 mL Silwet L-77) to obtain the root surface microbiome. The microbial community was separated by thoroughly centrifuging at a high speed to remove the root surface soil and then filtered through a 100 mm mesh cell strainer to remove plant debris and large soil aggregates. The filtrate was centrifuged at 5000 g for 15 min and collected in a new 50 mL centrifuge tube. Then, 1 mL PBS buffer was added to the centrifuge tube to suspend the pellet containing microorganisms, and then the samples were frozen in liquid nitrogen and stored at −80 °C. All the experiments were performed with three replicates per soil group.

PowerSoil^®^ DNA Isolation Kit (MoBio Laboratories, Carlsbad, CA, USA) was used to extract rhizosphere soils genomic DNA and 250 mg of soil was used according to the manufacturer’s instructions.

### 4.4. 16S rRNA Gene Sequencing and High-Throughput Sequencing

Extractive DNA quality and concentrations were checked by 0.8% agarose gel electrophoresis and ultraviolet spectrophotometry [13]. High quality DNA samples were used for bacterial 16S rRNA gene amplification by Beijing SinoGenoMax (Beijing, China). The specific primers 341F (forward primer, 5′-CCTACGGGNGGCWGCAG-3′) and 805R (reverse primer, 5′-GACTACNVGGGTATCTAATCC-3′) with barcode were used for bacterial 16S rRNA gene tags (V3 and V4 region) amplification [45]. PCR amplification was carried out with 2 × Phanta Max Master Mix (P515, Vazyme, Nanjing, China), with three phases of PCR program: A first phase consisting of 95 °C for 5 min, 7 cycles of 95 °C for 45 s, 65 °C for 1 min (decreasing at 2 °C/cycle), and 72 °C for 90 s, followed by a second phase consisting of 30 cycles of 95 °C for 45 s, 50 °C for 30 s, and 72 °C for 90 s, and then a final extension at 72 °C for 10 min. PCR products were checked by 0.8% agarose gel electrophoresis and further purified with Qiagen Gel Extraction Kit (Qiagen, Beijing, China). Sequencing libraries were produced by using TruSeq^®^ DNA PCR-Free Sample Preparation Kit (Illumina, San Diego, CA, USA) following manufacturer’s instructions. The library was sequenced on an Illumina HiSeq 2500 platform and 250 bp paired-end reads were generated. Raw reads were submitted to NCBI SRA under accession SUB5448618 and bioproject PRJNA532487.

### 4.5. Bioinformatics Analysis

The paired-end reads were merged with custom scripts (https://github.com/RiceMicrobiome/Edwards-et-al.-2014/tree/master/sequencing_scripts, accessed on: 26 November 2018) and assembled into single sequences with PANDAseq. Raw tags were preliminary screened to obtain the high-quality clean tags according to the QIIME software v1.8.0 quality controlled process. Clean tags were compared with the reference Gold database (http://drive5.com/uchime/uchime_download.html, accessed on: 14 December 2018) using UCHIME algorithm to detect chimera sequences [18]. Chimera sequences were discarded and effective tags finally obtained by using USEARCH (v5.2.236, http://www.drive5.com/usearch/, accessed on: 17 December 2018) [46]. OTU was defined as the sequence of one or more samples based on a sequence similarity threshold set. Sequences with ≥97% similarity value were assigned to the same OTUs, which is roughly equal to the sequence difference among taxonomic species in genetic diversity analysis based on 16S rRNA genes [47]. We pick a representative sequences for each OTU and use the RDP classifier to annotate taxonomic information for each representative sequence. To identify differences of microbial communities among the four soil groups, Venn diagrams were plotted with the VennDiagram package [48].

### 4.6. Alpha and Beta Diversity Analysis

In-house Perl scripts were used to analyze alpha (within samples) and beta (among samples) diversity. In order to compute alpha diversity, we analyzed the OTU table and calculated six indexes: Sobs, Chao1, ace, Shannon, Simpson and Coverage (access on 2 March 2019). Sobs exhibits the numbers of OTUs in peanut rhizosphere; Chao1 and ace indices can reflect the community abundance; Shannon and Simpson indices exhibit community diversity; Coverage can reflect the sequencing depth to observe community richness. In addition, rank abundance can estimate the species evenness, and rarefaction curves evaluating the species richness and depth of sequence was made using QIIME [49]. Furthermore, the species accumulation curve was used to reflect whether the sequence depth is sufficient to estimate community richness [50]. The rank abundance curve representing the numbers of abundant and rare OTUs can reflect the species abundance and evenness (http://en.wikipedia.org/wiki/Rank_abundance_curve, accessed on: 12 March 2019) [51]. Each OTU representative sequence in our study was used for taxonomic identification of each soil sample at different classification levels (phylum, class, order, family, and genus).

Beta diversity analysis was conducted to examine the similarity of the community structure among different soil samples. The PCA and PCoA analysis were performed on the community composition structure at the genus level to explore the similarities or dissimilarities among the four soil groups, which was applied to reduce the dimension of the original variables using the QIIME software package [52]. Cluster analysis mainly refers to the hierarchical clustering analysis method using any distance to evaluate the similarity among the soil groups (access on 16 March 2019) [53]. 

### 4.7. LEfSe and COG Analysis

LEfSe as an algorithm for high-dimensional biomarkers was used for the quantitative analysis of biomarkers within different soil groups. This method was designed to analyze data in which the number of species was much higher than the number of samples and to provide biological class explanations to establish statistical significance, biological consistency, and effect-size estimation of predicted biomarkers [54]. LEfSe analysis was performed on the website http://huttenhower.sph.harvard.edu/galaxy (accessed on: 21 March 2019). The differential features were identified on the OTU level. LEfSe analysis was performed with the alpha value for the factorial Kruskal–Wallis test among classes was <0.05 and the threshold on the logarithmic LDA score for discriminative features was >3.0 [55].

PICRUSt10 program was used to predict functional features of microbial community via 16S rRNA gene amplification based high-throughput sequencing data in the context of the COG database according to the previous study [56].

### 4.8. Statistical Tests

A permutation-based PERMANOVA (permutational multivariate analysis of variance) analysis was performed using QIIME software and 999 displacement tests to determine whether the differences between the drought-treated and untreated soil groups were statistically significant. The heat maps visualization with hierarchical clustering of the top 50 most abundant genera were generated using R-package, gplots (version 3.3.1) and color-coded by row z-scores [57].

### 4.9. Availability of Data and Materials 

All relevant data and materials that support the findings of this study are available from the corresponding author upon request.

## Supplementary Materials

Supplementary materials can be found at https://www.mdpi.com/1422-0067/20/9/2265/s1. Supplementary Figure S1: Overall sequence information of microbial communities in peanut rhizosphere. Supplementary Figure S2: Circos showing the similarity and dissimilarity of microbial community among four soil groups. Supplementary Figure S3: Beta diversity analysis showing the similarity of the community structure among different soil samples. Supplementary Figure S4: The circular phylogenetic tree showing the phylogenetic relationship of microbial community. Supplementary Figure S5: LEfSe Bar showing specific phylotypes of peanut rhizosphere responding to drought stress. Supplementary Table S1: The sequencing quantity of each soil sample after removing the doubtful sequences. Supplementary Table S2: Different alpha diversity indexes analysis of microbial community. Supplementary Table S3: Distribution and abundance of taxa at the phylum level. Supplementary Table S4: Distribution and abundance of taxa at the class level. Supplementary Table S5: Distribution and abundance of taxa at the genus level. Supplementary Excel S1: All the taxa detected at the genus level in peanut rhizosphere.

## Abbreviations

CR	Control rhizosphere
SDR	Seedling stage drought-treated rhizosphere
PDR	Flowering stage drought-treated rhizosphere
FDR	Podding stage drought-treated rhizosphere
QIIME	Quantitative Insights into Microbial Ecology
OTUs	Operational taxonomic units
PCA	Principal component analysis
PCoA	Principal co-ordinates analysis
LDA	Linear discriminant analysis
LEfSe	LDA effect size
COG	Cluster of Orthologous Groups

## References

[1] Wang F.P., Wang X.F., Zhang J., Ma F., Hao Y.J. (2018). MdMYB58 Modulates Fe Homeostasis by Directly Binding to the MdMATE43 Promoter in Plants. Plant Cell Physiol..

[2] Sui N., Wang Y., Liu S., Yang Z., Wang F., Wan S. (2018). Transcriptomic and Physiological Evidence for the Relationship between Unsaturated Fatty Acid and Salt Stress in Peanut. Front. Plant Sci..

[3] Wan L., Wu Y., Huang J., Dai X., Lei Y., Yan L., Jiang H., Zhang J., Varshney R.K., Liao B. (2014). Identification of ERF genes in peanuts and functional analysis of AhERF008 and AhERF019 in abiotic stress response. Funct. Integr. Genom..

[4] Cuc L.M., Mace E.S., Crouch J.H., Quang V.D., Long T.D., Varshney R.K. (2008). Isolation and characterization of novel microsatellite markers and their application for diversity assessment in cultivated groundnut (Arachis hypogaea). BMC Plant Biol..

[5] Krishna G., Singh B.K., Kim E.K., Morya V.K., Ramteke P.W. (2015). Progress in genetic engineering of peanut (Arachis hypogaea L.)—A review. Plant Biotechnol. J..

[6] Zhang X., Lu G., Long W., Zou X., Li F., Nishio T. (2014). Recent progress in drought and salt tolerance studies in Brassica crops. Breed Sci..

[7] Sarkar T., Thankappan R., Kumar A., Mishra G.P., Dobaria J.R. (2016). Stress Inducible Expression of AtDREB1A Transcription Factor in Transgenic Peanut (Arachis hypogaea L.) Conferred Tolerance to Soil-Moisture Deficit Stress. Front. Plant Sci..

[8] Boudsocq M., Lauriere C. (2005). Osmotic signaling in plants: Multiple pathways mediated by emerging kinase families. Plant Physiol..

[9] Sahebi M., Hanafi M.M., Rafii M.Y., Mahmud T.M.M., Azizi P., Osman M., Abiri R., Taheri S., Kalhori N., Shabanimofrad M. (2018). Improvement of Drought Tolerance in Rice (Oryza sativa L.): Genetics, Genomic Tools, and the WRKY Gene Family. Biomed. Res. Int..

[10] Hoekstra F.A., Golovina E.A., Buitink J. (2001). Mechanisms of plant desiccation tolerance. Trends Plant Sci..

[11] Sun M., Xu Y., Huang J., Jiang Z., Shu H., Wang H., Zhang S. (2017). Global Identification, Classification, and Expression Analysis of MAPKKK genes: Functional Characterization of MdRaf5 Reveals Evolution and Drought-Responsive Profile in Apple. Sci. Rep..

[12] Xu Y., Zheng X., Song Y., Zhu L., Yu Z., Gan L., Zhou S., Liu H., Wen F., Zhu C. (2018). NtLTP4, a lipid transfer protein that enhances salt and drought stresses tolerance in Nicotiana tabacum. Sci. Rep..

[13] Ullah A., Akbar A., Luo Q., Khan A.H., Manghwar H., Shaban M., Yang X. (2018). Microbiome Diversity in Cotton Rhizosphere Under Normal and Drought Conditions. Microb. Ecol..

[14] Ullah A., Sun H., Yang X., Zhang X. (2017). Drought coping strategies in cotton: Increased crop per drop. Plant Biotechnol. J..

[15] He A.L., Niu S.Q., Zhao Q., Li Y.S., Gou J.Y., Gao H.J., Suo S.Z., Zhang J.L. (2018). Induced Salt Tolerance of Perennial Ryegrass by a Novel Bacterium Strain from the Rhizosphere of a Desert Shrub Haloxylon ammodendron. Int. J. Mol. Sci..

[16] Mateus J.R., Marques J.M., Dal’Rio I., Vollu R.E., Coelho M.R.R., Seldin L. (2019). Response of the microbial community associated with sweet potato (Ipomoea batatas) to Bacillus safensis and Bacillus velezensis strains. Antonie Van Leeuwenhoek.

[17] Mendes R., Garbeva P., Raaijmakers J.M. (2013). The rhizosphere microbiome: Significance of plant beneficial, plant pathogenic, and human pathogenic microorganisms. FEMS Microbiol. Rev..

[18] Geng L.L., Shao G.X., Raymond B., Wang M.L., Sun X.X., Shu C.L., Zhang J. (2018). Subterranean infestation by Holotrichia parallela larvae is associated with changes in the peanut (Arachis hypogaea L.) rhizosphere microbiome. Microbiol. Res..

[19] Dennis P.G., Miller A.J., Hirsch P.R. (2010). Are root exudates more important than other sources of rhizodeposits in structuring rhizosphere bacterial communities?. FEMS Microbiol. Ecol..

[20] Bai Y., Muller D.B., Srinivas G., Garrido-Oter R., Potthoff E., Rott M., Dombrowski N., Munch P.C., Spaepen S., Remus-Emsermann M. (2015). Functional overlap of the Arabidopsis leaf and root microbiota. Nature.

[21] Cui J., Li Y., Wang C., Kim K.S., Wang T., Liu S. (2018). Characteristics of the rhizosphere bacterial community across different cultivation years in saline-alkaline paddy soils of Songnen Plain of China. Can. J. Microbiol..

[22] Rodrigues R.R., Pineda R.P., Barney J.N., Nilsen E.T., Barrett J.E., Williams M.A. (2015). Plant Invasions Associated with Change in Root-Zone Microbial Community Structure and Diversity. PLoS ONE.

[23] Evelin H., Kapoor R., Giri B. (2009). Arbuscular mycorrhizal fungi in alleviation of salt stress: A review. Ann. Bot..

[24] Yang J., Kloepper J.W., Ryu C.M. (2009). Rhizosphere bacteria help plants tolerate abiotic stress. Trends Plant Sci..

[25] Yang Y., Wang N., Guo X., Zhang Y., Ye B. (2017). Comparative analysis of bacterial community structure in the rhizosphere of maize by high-throughput pyrosequencing. PLoS ONE.

[26] Bulgarelli D., Garrido-Oter R., Munch P.C., Weiman A., Droge J., Pan Y., McHardy A.C., Schulze-Lefert P. (2015). Structure and function of the bacterial root microbiota in wild and domesticated barley. Cell Host Microbe.

[27] Qiao Q., Wang F., Zhang J., Chen Y., Zhang C., Liu G., Zhang H., Ma C., Zhang J. (2017). The Variation in the Rhizosphere Microbiome of Cotton with Soil Type, Genotype and Developmental Stage. Sci. Rep..

[28] Lundberg D.S., Lebeis S.L., Paredes S.H., Yourstone S., Gehring J., Malfatti S., Tremblay J., Engelbrektson A., Kunin V., Del Rio T.G. (2012). Defining the core Arabidopsis thaliana root microbiome. Nature.

[29] Naylor D., DeGraaf S., Purdom E., Coleman-Derr D. (2017). Drought and host selection influence bacterial community dynamics in the grass root microbiome. ISME J..

[30] Marques J.M., da Silva T.F., Vollu R.E., Blank A.F., Ding G.C., Seldin L., Smalla K. (2014). Plant age and genotype affect the bacterial community composition in the tuber rhizosphere of field-grown sweet potato plants. FEMS Microbiol. Ecol..

[31] Inceoglu O., Salles J.F., van Overbeek L., van Elsas J.D. (2010). Effects of plant genotype and growth stage on the betaproteobacterial communities associated with different potato cultivars in two fields. Appl. Environ. Microbiol..

[32] Bullerjahn G.S., Post A.F. (2014). Physiology and molecular biology of aquatic cyanobacteria. Front. Microbiol..

[33] Singh H. (2018). Desiccation and radiation stress tolerance in cyanobacteria. J. Basic Microbiol..

[34] DeBruyn J.M., Nixon L.T., Fawaz M.N., Johnson A.M., Radosevich M. (2011). Global biogeography and quantitative seasonal dynamics of Gemmatimonadetes in soil. Appl. Environ. Microbiol..

[35] Doolotkeldieva T., Konurbaeva M., Bobusheva S. (2018). Microbial communities in pesticide-contaminated soils in Kyrgyzstan and bioremediation possibilities. Environ. Sci. Pollut. Res. Int..

[36] Jeske O., Surup F., Ketteniss M., Rast P., Forster B., Jogler M., Wink J., Jogler C. (2016). Developing Techniques for the Utilization of Planctomycetes As Producers of Bioactive Molecules. Front. Microbiol..

[37] Fozo E.M., Quivey R.G. (2004). Shifts in the membrane fatty acid profile of Streptococcus mutans enhance survival in acidic environments. Appl. Environ. Microbiol..

[38] Numan M., Bashir S., Khan Y., Mumtaz R., Shinwari Z.K., Khan A.L., Khan A., Al-Harrasi A. (2018). Plant growth promoting bacteria as an alternative strategy for salt tolerance in plants: A review. Microbiol. Res..

[39] Mirete S., Mora-Ruiz M.R., Lamprecht-Grandio M., de Figueras C.G., Rossello-Mora R., Gonzalez-Pastor J.E. (2015). Salt resistance genes revealed by functional metagenomics from brines and moderate-salinity rhizosphere within a hypersaline environment. Front. Microbiol..

[40] Fu C., Liu X.X., Yang W.W., Zhao C.M., Liu J. (2016). Enhanced salt tolerance in tomato plants constitutively expressing heat-shock protein in the endoplasmic reticulum. Genet. Mol. Res..

[41] Guan P., Wang J., Li H., Xie C., Zhang S., Wu C., Yang G., Yan K., Huang J., Zheng C. (2018). SENSITIVE TO SALT1, An Endoplasmic Reticulum-Localized Chaperone, Positively Regulates Salt Resistance. Plant Physiol..

[42] Wu L., Wang J., Wu H., Chen J., Xiao Z., Qin X., Zhang Z., Lin W. (2018). Comparative Metagenomic Analysis of Rhizosphere Microbial Community Composition and Functional Potentials under Rehmannia glutinosa Consecutive Monoculture. Int. J. Mol. Sci..

[43] Ding H., Zhang Z., Kang T., Dai L., Ci D., Qin F., Song W. (2017). Rooting traits of peanut genotypes differing in drought tolerance under drought stress. Int. J. Plant Prod..

[44] Bulgarelli D., Rott M., Schlaeppi K., Ver Loren van Themaat E., Ahmadinejad N., Assenza F., Rauf P., Huettel B., Reinhardt R., Schmelzer E. (2012). Revealing structure and assembly cues for Arabidopsis root-inhabiting bacterial microbiota. Nature.

[45] Herlemann D.P., Labrenz M., Jurgens K., Bertilsson S., Waniek J.J., Andersson A.F. (2011). Transitions in bacterial communities along the 2000 km salinity gradient of the Baltic Sea. ISME J..

[46] Caporaso J.G., Kuczynski J., Stombaugh J., Bittinger K., Bushman F.D., Costello E.K., Fierer N., Pena A.G., Goodrich J.K., Gordon J.I. (2010). QIIME allows analysis of high-throughput community sequencing data. Nat. Methods.

[47] Blaxter M., Mann J., Chapman T., Thomas F., Whitton C., Floyd R., Abebe E. (2005). Defining operational taxonomic units using DNA barcode data. Philos. Trans. R. Soc. B Biol. Sci..

[48] Chen H., Boutros P.C. (2011). VennDiagram: A package for the generation of highly-customizable Venn and Euler diagrams in R. BMC Bioinform..

[49] Chen B., Teh B.S., Sun C., Hu S., Lu X., Boland W., Shao Y. (2016). Biodiversity and Activity of the Gut Microbiota across the Life History of the Insect Herbivore Spodoptera littoralis. Sci. Rep..

[50] Maughan H., Wang P.W., Diaz Caballero J., Fung P., Gong Y., Donaldson S.L., Yuan L., Keshavjee S., Zhang Y., Yau Y.C. (2012). Analysis of the cystic fibrosis lung microbiota via serial Illumina sequencing of bacterial 16S rRNA hypervariable regions. PLoS ONE.

[51] Bates S.T., Clemente J.C., Flores G.E., Walters W.A., Parfrey L.W., Knight R., Fierer N. (2013). Global biogeography of highly diverse protistan communities in soil. ISME J..

[52] Wang Y., Sheng H.F., He Y., Wu J.Y., Jiang Y.X., Tam N.F., Zhou H.W. (2012). Comparison of the levels of bacterial diversity in freshwater, intertidal wetland, and marine sediments by using millions of illumina tags. Appl. Environ. Microbiol..

[53] Jin S., Zhao D., Cai C., Song D., Shen J., Xu A., Qiao Y., Ran Z., Zheng Q. (2017). Low-dose penicillin exposure in early life decreases Th17 and the susceptibility to DSS colitis in mice through gut microbiota modification. Sci. Rep..

[54] Segata N., Izard J., Waldron L., Gevers D., Miropolsky L., Garrett W.S., Huttenhower C. (2011). Metagenomic biomarker discovery and explanation. Genome Biol..

[55] Zhang C., Li S., Yang L., Huang P., Li W., Wang S., Zhao G., Zhang M., Pang X., Yan Z. (2013). Structural modulation of gut microbiota in life-long calorie-restricted mice. Nat. Commun..

[56] Langille M.G., Zaneveld J., Caporaso J.G., McDonald D., Knights D., Reyes J.A., Clemente J.C., Burkepile D.E., Vega Thurber R.L., Knight R. (2013). Predictive functional profiling of microbial communities using 16S rRNA marker gene sequences. Nat. Biotechnol..

[57] Zuo Y., Xie W., Pang Y., Li T., Li Q., Li Y. (2017). Bacterial community composition in the gut content of Lampetra japonica revealed by 16S rRNA gene pyrosequencing. PLoS ONE.

